# Antifreezing Proton Zwitterionic Hydrogel Electrolyte via Ionic Hopping and Grotthuss Transport Mechanism toward Solid Supercapacitor Working at −50 °C

**DOI:** 10.1002/advs.202201679

**Published:** 2022-07-26

**Authors:** Weigang Sun, Zhen Xu, Congde Qiao, Bingxi Lv, Ligang Gai, Xingxiang Ji, Haihui Jiang, Libin Liu

**Affiliations:** ^1^ School of Chemistry and Chemical Engineering State Key Laboratory of Biobased Material and Green Papermaking Qilu University of Technology (Shandong Academy of Sciences) Jinan 250353 China

**Keywords:** hydrogel electrolyte, low temperature, proton conductivity, supercapacitor, sulphuric acid

## Abstract

Hydrogel electrolyte is widely used in solid energy storage devices because of its high ionic conductivity, environmental friendliness, and non‐leakage property. However, hydrogel electrolyte is not resistant to freezing. Here, a high proton conductive zwitterionic hydrogel electrolyte with super conductivity of 1.51 mS cm^–1^ at −50 °C is fabricated by random copolymerization of acrylamide and zwitterionic monomer in the presence of 1 m H_2_SO_4_ and ethylene glycol (EG). The antifreezing performance and low temperature conductivity are ascribed to hydrogen bonds and ionic bonds between the components and water molecules in the system and can be tuned by changing the monomer ratio and EG contents. The proton hopping migration on the ionic group of the polymer chains and Grotthuss proton transport mechanism are responsible for the high proton conductivity while Grotthuss transport is dominated at the glassy state of the polymer chains. The electrolyte‐assembled supercapacitor (SC) offers high specific capacitance of 93.5 F g^–1^ at 25 °C and 62.0 F g^–1^ at −50 °C with a capacitance retention of 91.1% and 81.5% after 10 000 cycles, respectively. The SC can even work at −70 °C. The electrolyte outperforms most reported antifreezing hydrogel electrolytes and has high potential in low‐temperature devices.

## Graphical abstract

A serial of proton antifreezing hydrogel electrolytes are fabricated. An extreme low‐temperature conductivity of 1.51 mS cm^–1^ at −50 °C is obtained. The proton transfer is facilitated by hopping on the zwitterionic group and Grotthuss proton conductivity mechanism. The electrolyte‐assembled supercapacitor demonstrates good electrochemical performance at low temperature of −50 °C.

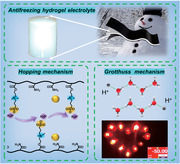

## 1 Introduction

Low temperature energy storage devices are essential for extreme cold climate such as aerospace exploration, polar exploration and high‐altitude activities. As an important part of energy storage devices, the freezing resistance of electrolytes directly affects the low‐temperature performance of energy storage devices. Although a large number of studies are reported on non‐aqueous electrolytes with low melting point,^[^ ^1^, ^2^, ^3^, ^4^, ^5^ ^]^ such as liquefied gas,^[^ ^2^ ^]^ fluorinated solvent,^[^ ^3^ ^]^ and ethyl acetate,^[^ ^4^ ^]^ these non‐aqueous electrolytes are usually costly, leaky, flammable and toxic, restricting their applications. In contrast, water‐based electrolytes perform well in terms of low cost, non‐flammability and non‐toxicity, etc. Specifically, as a water‐based solid electrolyte, hydrogel electrolytes are the solution to the problem of easy leakage of liquid water‐based electrolytes, and therefore, hydrogel electrolytes are attracting more and more attention in the field of large‐scale energy storage.^[^ ^6^, ^7^ ^]^ However, the large amount of free water in the hydrogel electrolyte inevitably freezes at subzero temperatures, causing the decrease of ionic conductivity and loss of flexibility of hydrogels.

At present, there are three main strategies to solve the problem of freezing of hydrogel electrolyte. One is by using salts as inhibitors of water freezing.^[^ ^8^, ^9^, ^10^, ^11^, ^12^ ^]^ In these systems, only high concentration of salts can reduce the freezing point. For example, 30 wt% CaCl_2_ can reduce the freezing point of the hydrogel system to −57 °C, and the low salt concentration (10 wt% CaCl_2_) is only reduced to −7 °C.^[^ ^10^ ^]^ In these antifreezing hydrogels, the salts with high concentration are corrosive to energy storage devices. Another strategy is by using ionic liquids to improve the antifreezing performance of hydrogels.^[^ ^13^, ^14^, ^15^ ^]^ For example, Zang et al. developed an ionic liquid‐based supercapacitor that can work at −40 °C.^[^ ^13^ ^]^ However, the high viscosity and inferior ionic conductivity of ionic liquids at low temperatures are also not conducive to wide applications.^[^ ^16^, ^17^, ^18^ ^]^ The third strategy is by introducing organic liquids into hydrogels to inhibit the formation of ice crystals. Commonly used organic liquids are dimethyl sulfoxide,^[^ ^19^, ^20^ ^]^ EG,^[^ ^21^, ^22^, ^23^, ^24^ ^]^ glycerol,^[^ ^25^, ^26^ ^]^ acetonitrile,^[^ ^27^, ^28^ ^]^ and so on. Among these strategies, EG is widely used as engine coolant in industry because of its low price and environmental friendliness.

The ideal antifreezing hydrogel electrolyte needs to meet various functionalities in an equilibrium system such as low freezing point, environmental friendliness, excellent low temperature ionic conductivity and mechanical flexibility. Proton, compared with heavy mass and large radius of metal ions, is an ideal conductive ion owing to its small size, wide availability, and negligible cost. Up to now, great progress has been made on aqueous proton electrolytes^[^ ^29^, ^30^, ^31^ ^]^ and proton hydrogel electrolyte.^[^ ^32^, ^33^, ^34^ ^]^ However, these proton electrolytes are either not freezing tolerant or antifreezing through high concentration of proton acid. For example, a high concentration of H_2_SO_4_ (5 m), which may be corrosive to the electrode, was applied in antifreezing aqueous Pb‐quinone battery.^[^ ^31^ ^]^ Therefore, developing proton hydrogel electrolyte by using a low concentration of proton acid with excellent low‐temperature conductivity and mechanical flexibility is highly demanded.

**Figure 1:**
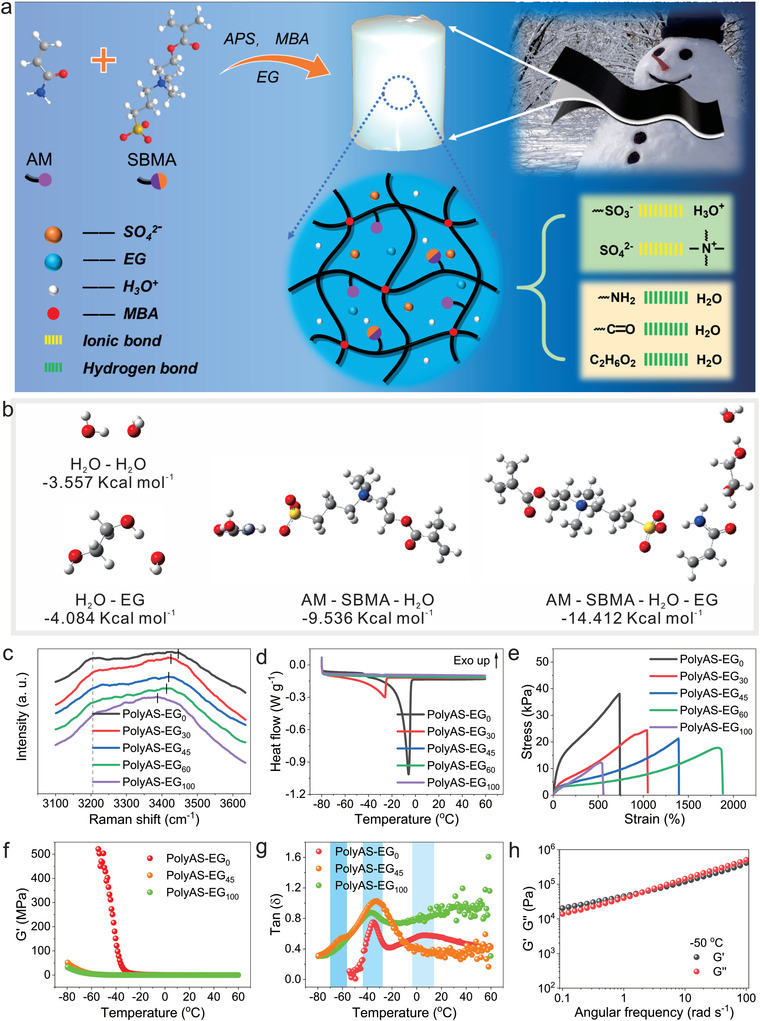
a) Schematic diagram of fabrication process of antifreezing poly(AM‐ SBMA) hydrogel electrolyte with internal ionic and hydrogen bonding interaction. b) Interaction energies of different components in the electrolyte. c) Raman spectra of water molecules in the electrolytes. d) DSC curves of the electrolytes with different EG contents. e) Stress–strain curves of different electrolytes. f) Storage modulus (G′) of the different electrolytes at different temperatures. g) Loss factor tan*δ* of the electrolytes at different temperatures. h) Frequency dependence of G′ and loss modulus (G″) of polyAS‐EG_45_ at low temperature of −50 °C.

In this work, antifreezing proton hydrogel electrolytes are prepared by one‐step radical polymerization of acrylamide (AM) and zwitterionic [(methacryloyloxy)ethyl]dimethyl‐(3‐sulfopropyl)ammonium hydroxide (SBMA) in the presence of 1 m H_2_SO_4_ and EG with *N*,*N*‐methylenebisacrylamide (MBA) used as a cross‐linker (**Figure** **1**a). Due to the multiple ionic bonds and hydrogen bonds in the system, the electrolytes show excellent mechanical properties and antifreezing performance. The low‐temperature conductivity of the hydrogel electrolyte can achieve as high as 1.51 mS cm^−1^ at −50 °C. The proton hopping migration on the ionic group of the SBMA and Grotthuss proton transport mechanism are responsible for the high conductivity of the proton while Grotthuss mechanism is dominated at the glassy state of the polymer chains in the low temperature environments. The supercapacitor assembled with electrolyte possesses high capacitance of 62.0 F g^–1^ at −50 °C with capacitance retention of 81.5% after 10000 cycles and even can be used at very low temperature of ‐70 °C.

## 2 Results and Discussion

SBMA has been proved by others^[^ ^35^ ^]^ and our group^[^ ^36^ ^]^ to be beneficial to the dissociation of metal salts, previously. In this work, H_2_SO_4_ is used as the conductive media and SO_3_^−^ of the SBMA has been considered to facilitate proton conductivity. Density functional theory (DFT) calculations is first used to prove the favorable proton conductivity of SBMA. Before addition of H_2_SO_4_ into the poly(AM‐SBMA) hydrogels, SBMA with the anions and cations can interact with each other to form inner salts with optimum configuration (*E*_SBMA‐SBMA_: −1.378 kcal mol^–1^). After introduction of H_2_SO_4_, the interaction energy of –SO_3_^–^H^+^ (*E*_SBMA‐H+_: −172.967 kcal mol^–1^) is lower than that of –HSO_4_^–^H^+^ (*E*_HSO4‐H+_: −168.493 kcal mol^–1^) (Figure S1, Supporting Information), indicating that SBMA is favorable to the dissociation of sulfuric acid. The measured ionic conductivity of poly(AM‐SBMA) electrolytes without EG also reveals that high content of SBMA is benefit to conductivity of the systems (Figure S2a, Supporting Information). However, the more amount of SBMA is, the smaller the modulus of the hydrogel electrolyte is (Figure S2b, Supporting Information). Considering ionic conductivity and mechanical properties, the electrolyte with the molar ratio of AM : SBMA = 3 : 1 is selected to study the antifreezing performance by adding different amount of EG (hereinafter abbreviated as polyAS‐EGx, x represents the volume percentage of EG). Regardless of the EG content, all the gel electrolytes show good solid‐state behaviors (Figure S3, Supporting Information).

The essence of water freezing is the phase transition from disordered water molecules to ordered ice driven by hydrogen bonds.^[^ ^37^ ^]^ EG, as both hydrogen bond acceptor and donor, can effectively compete with hydrogen and oxygen in water molecules, thereby destroying the original hydrogen bonding network between water molecules and effectively reducing the freezing point of aqueous solution. The interaction energy of EG and water is −4.084 kcal mol^–1^, lower than that of water‐water system (*E*_H2O‐H2O_: −3.557 kcal mol^–1^) and EG‐EG system (*E*_EG‐EG_: −3.498 kcal mol^–1^) (Figure 1b, Figure S4, Supporting Information), indicating the more stable hydrogen bonds in EG‐water systems. In addition, the interaction energy of segments of poly(AM‐SBMA) chains with EG‐water mixtures (*E*_AM‐SBMA‐EG‐H2O_: −14.412 kcal mol^–1^) is much lower than that of polymer chains with pure water (*E*_AM‐SBMA‐H2O_: −9.536 kcal mol^–1^) or with pure EG (*E*_AM‐SBMA‐EG_: −10.069 kcal mol^–1^) (Figure 1b, Figure S4, Supporting Information), demonstrating that the EG–water mixtures can form more stable hydrogel networks than pure water or pure EG, and thus are favorable to restricting the freezing of water.

Raman spectra also confirm the hydrogen bonding formation among water and EG molecules. As shown in Figure 1c, two broad peaks are centered at 3205 and 3445 cm^–1^ for polyAS‐EG_0_, which are attributed to O–H stretching vibration of hydrogen bonds of water molecules. After addition of EG, the peak at 3445 cm^–1^ is blue‐shifted to 3424 cm^–1^ for polyAS‐EG_30_ and further blue‐shifted to 3386 cm^–1^ for polyAS‐EG_100_,^[^ ^38^ ^]^ indicating that the hydrogen–bonding interactions among water molecules have been weakened in the EG–water system, thus inhibiting the formation of ice crystals at low temperature. Differential scanning calorimetry (DSC) is used to observe the effect of different EG contents on the freezing point of the electrolytes. As shown in Figure 1d, when the EG content is 0 and 30 vol%, the freezing point of polyAS‐EG_0_ and polyAS‐EG_30_ is −3 and −22.9 °C, respectively. When the EG content is 45, 60, and 100 vol%, the endothermic peak is not observed in the DSC curves, indicating that there is no change in heat flow in the electrolyte.

Different content of EG also affects the mechanical properties of the electrolytes. As shown in Figure 1e, with the increase of EG, the stress of electrolytes decreases and the strain increases. For example, the stress and strain change from 38.1 kPa and 736% for polyAS‐EG_0_ to 17.8 kPa and 1840% for polyAS‐EG_60_. To further understand the inner structure of the electrolytes at low temperature, temperature sweep of rheology is performed. The temperature sweep shows that the G’ of polyAS‐EG_0_ sharply increases at about −40 °C, indicating the froze of electrolyte. In contrast, the G′ of polyAS‐EG_45_ and polyAS‐EG_100_ is extraordinary stable at a wide temperature range of −60 to 60 °C (Figure 1f). In addition, the tan*δ* curves reveal that polyAS‐EG_0_ shows a wide peak at about 0^ ^°C due to the freezing of free water (Figure 1g), which is consistent with DSC results. Surprisingly, a solvent‐independent peak appears at −38 °C for all three electrolytes, which is considered to be the glass transition temperature (*T*_g_) of poly(AM‐SBMA) chains in the electrolyte. Also, polyAS‐EG_45_ electrolyte possesses a weak shoulder centered at −60 °C while polyAS‐EG_0_ and polyAS‐EG_100_ do not. This peak may be caused by crystallization of partially bound water. In polyAS‐EG_45_ electrolyte at −60 °C, it should be a two‐phase system containing trace ice. The two‐phase system is further confirmed by frequency scanning at a low temperature of −50 °C. With the increase of frequency, the G″ is gradually higher than the G′ (Figure 1h), indicating the phase transition of electrolyte from solid to liquid. Although trace ice exists in the electrolyte, the polyAS‐EG_45_ electrolyte still shows flexibility at low temperature of −60 °C (Figure S5, Supporting Information).

**Figure 2:**
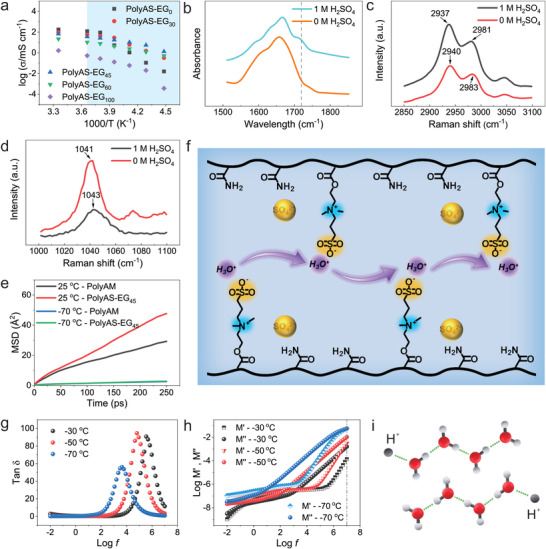
a) Temperature dependence of ionic conductivity of different electrolytes. b) FTIR spectra of the bending vibration of flanking waters of the hydronium ions (H_3_O^+^). Raman spectra of c) –N^+^(CH_3_)_2_ and d) –SO_3_^–^ vibration of SBMA for the electrolytes with and without H_2_SO_4_. e) MSD of H_3_O^+^ in polyAM and polyAS‐EG_45_ electrolytes at different temperatures. f) Proposed proton hopping on –SO_3_^−^ site of SBMA. Broadband dielectric spectroscopy of the polyAS‐EG_45_. g) Electrode polarization process and h) dielectric modulus (M′) and dielectric loss modulus (M″) of polyAS‐EG_45_ as a function of frequency at different temperatures. i) Schematic of the Grotthuss mechanism in the electrolytes.

Besides the antifreezing performance, our hydrogel electrolytes also exhibit excellent conductivity at low temperatures. The conductivity of the electrolytes with different EG contents measured at different temperatures is shown in **Figure** **2**a. With the increase of EG content, the conductivity of the electrolytes first increases and then decreases, mainly because the increase of EG content is conductive to the frost resistance, but too high EG content is not favorable to the ion migration. The polyAS‐EG_45_ exhibits the highest ionic conductivity of 1.51 mS cm^−1^ at −50 °C (**Table** **1**), which is ten times more than that of antifreezing aqueous 2 m NaClO_4_/DMSO electrolyte (0.11 mS cm^–1^ at −50 °C),^[^ ^19^ ^]^ further confirming the advantages of proton conduction. The super low temperature conductivity of our hydrogel electrolyte is also in the highest level among that of the reported antifreezing hydrogel electrolyte (Table S2, Supporting Information) and even one hundred time that of montmorillonite/poly(vinyl alcohol) hydrogel electrolyte with 2 m H_2_SO_4_.^[^ ^33^ ^]^ It is noted that under subzero temperature range the relationship between the ionic conductivity of electrolytes and the reciprocal of absolute temperature shows a linear relationship, obeying Arrhenius's law.^[^ ^39^ ^]^ The activation energy (*E*_a_) of different electrolytes are shown in Table 1. The lower the activation energy is, the more conducive to ion migration is. The lowest *E*_a_ of 0.258 eV was obtained for polyAS‐EG_45_ electrolyte, which is also consistent with the highest conductivity at −50 °C.

**Table 1:** Activation energy and conductivity of different electrolytes at −50 °C

Electrolytes	*σ*_‐50 _°_C_ [mS cm^–1^]	*E*_a_ [eV]
PolyAS‐EG_0_	0.016	0.945
PolyAS‐EG_30_	0.32	0.566
PolyAS‐EG_45_	1.51	0.258
PolyAS‐EG_60_	0.51	0.321
PolyAS‐EG_100_	0.0004	0.443

It is known that protons exist in aqueous solution in the form of hydronium ions (H_3_O^+^). In our work, a shoulder appears at 1720 cm^–1^ derived from the bending vibration of flanking waters of the H_3_O^+^ (Figure 2b), which indicates the presence of H_3_O^+^ in the systems.^[^ ^40^ ^]^ The high ionic conductivity may be due to hopping migration of H_3_O^+^ on sulfonate of SBMA, similar to the migration mechanism of metal ion in zwitterionic electrolytes.^[^ ^36^, ^41^, ^42^ ^]^ To prove this, the interaction of H_2_SO_4_ and polymer chains is measured. When H_2_SO_4_ is added to the hydrogel, the stretching vibration of ‐N^+^(CH_3_)_2_ peaked at 2983 and 2940 cm^–1^ is shifted to 2981 and 2937 cm^–1^, respectively, indicating the interaction between –SO_4_^2−^ and –N^+^(CH_3_)_2_ (Figure 2c). Also, the S═O stretching vibration in –SO_3_^–^ of SBMA is shifted from 1041 to 1043 cm^−1^ after addition of H_2_SO_4_ (Figure 2d). The changes of these characteristic peaks indicate that the static equilibrium of anions and cations in the SBMA is destroyed, and H^+^ and SO_4_^2–^ can easily overcome the electrostatic attraction by –SO_3_^–^ and –N^+^(CH_3_)_2_ group of SBMA. In order to further prove the hopping migration of H_3_O^+^ on sulfonate, root mean square ion displacement (MSD) has been carried out. As shown in Figure 2e, the displacement of H_3_O^+^ in polyAM and polyAS‐EG_45_ electrolyte reveals a linear relationship with the time interval and a larger slope for H_3_O^+^ in polyAS‐EG_45_ than that in polyAM electrolyte. This indicates a faster diffusion rate in polyAS‐EG_45_ electrolyte than that in polyAM electrolyte (Table S1, Supporting Information). Based on the above findings, the faster diffusion of H_3_O^+^ in polyAS‐EG_45_ and the lower interaction energy of –SO_3_^–^ and H^+^ demonstrate that the –SO_3_^−^ on the polymer chain should provide the channel for proton conduction^[^ ^43^ ^]^ and the H_3_O^+^ may hop through continuous complexation and decomplexation on the –SO_3_^−^ site of the poly(AM‐SBMA) chains at room temperature (Figure 2f), similar to the Li^+^ transport mechanism in zwitterionic polymer electrolytes.^[^ ^36^, ^42^ ^]^ However, at extreme low temperature of –70 °C, the MSD curves indicate that the diffusion of H_3_O^+^ in polyAM and polyAS‐EG_45_ has been significantly reduced (Figure 2e). This is consistent with the results of low temperature sweep of rheology (Figure 1g), where the *T*_g_ of the poly(AM‐SBMA) chains in the electrolytes is about −38 °C and the polyAS‐EG_45_ chains should be in glassy state at temperature lower than −38 °C. Thus, at such low temperature the hopping migration of protons on the molecular chain may be restricted. Therefore, the measured high ionic conductivity of 1.51 mS cm^−1^ at −50 °C indicates another effective proton transfer mechanism may be adopted at subzero temperature.

Generally speaking, protons usually adopt the typical Grotthuss transport mechanism where a given proton is transferred between two neighboring hydrogen bonded molecules in aqueous system.^[^ ^44^, ^45^ ^]^ We propose that the Grotthuss transport mechanisms should be adopted in our system and is dominated at subzero temperature. To prove this, broadband dielectric spectroscopy (BDS) was carried out in the external electric field with frequency interval of milli‐ to megahertz and a low temperature range of −30 to ‐70 °C. The electrode polarization process is shown in Figure 2g. The maximum frequency can be regarded as the beginning of the electrode polarization,^[^ ^46^ ^]^ and the structural/segmental relaxation time of the electrolyte can be obtained by the maximum frequency (1/2*π*f_mas_). With the decrease of temperature, the structural/segmental relaxation time of electrolyte increases from 4.61 × 10^−7^ s at −30 °C to 3.66 × 10^−5^ s at −70 °C (Figure 2g). In contrast, the conductivity relaxation time of the electrolyte at −70 °C is about 1.59 × 10^−8^ s, calculated from the reciprocal frequency at the crossing point of the electric modulus (M′) and the electric loss modulus (M″)^[^ ^47^ ^]^ (Figure 2h), which is much faster than the structural/segmental relaxation time at −70 °C and even faster than the structural/segmental relaxation time at −30 °C. The significantly faster time scale of the conductivity relaxation than that of structural/segmental relaxation is usually attributed to the proton transfer mechanism through the H‐bonded network (that is Grotthuss transport mechanisms) and does not require diffusion of entire molecular units.^[^ ^48^ ^]^ In addition, the proton conductivity of polyAS‐EG_45_ at −50 °C give an *E*_a_ of 0.258 eV, where an *E*_a_ value less than 0.4 eV is also considered to follow the Grotthuss theory,^[^ ^30^ ^]^ in which protons transfer through the neighboring water molecules via the bond vibrations of the H···O hydrogen bonds and H—O covalent bonds (Figure 2i).

**Figure 3:**
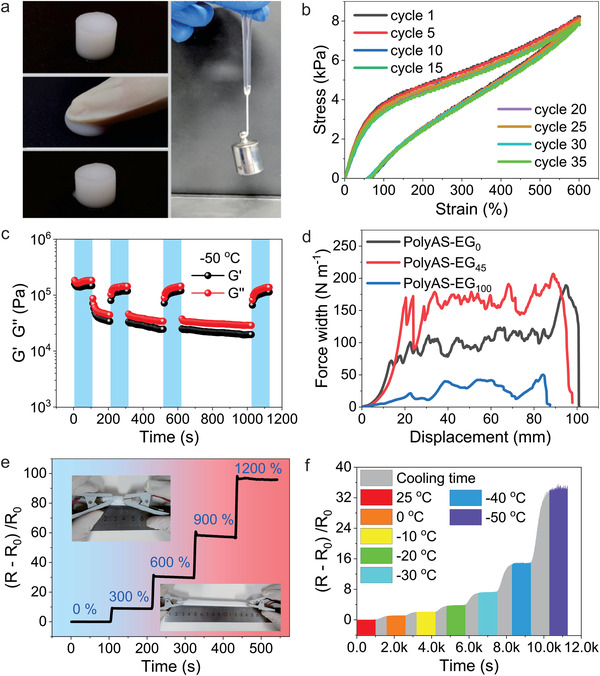
a) Photograph of the polyAS‐EG_45_ electrolyte under compression and loading of 50 g. b) Stress–strain cycles of polyAS‐EG_45_ electrolyte at room temperature. c) G′ and G″ of the polyAS‐EG_45_ electrolytes at −50 °C under an alternate strain of 1% for 100 s and 200% for 100, 200, and 400 s, respectively. d) Adhesion strength of the different electrolytes to carbon cloth substrate. e) Resistance response of polyAS‐EG_45_ electrolyte under different stretching strain (insets: photograph of electrolyte at stretched and released state, respectively). f) Resistance response of polyAS‐EG_45_ electrolyte at different cooling temperatures.

Besides the high conductivity of electrolyte, good mechanical properties at room temperature and subzero temperature are also the premise to ensure the long‐term application of electrolyte. The polyAS‐EG_45_ electrolyte can be compressed and released to its original state. Short rod of electrolyte with 6 mm in diameter can withstand 50 g loading (**Figure** **3**a). Stress–strain experiments of polyAS‐EG_45_ reveal that the curves almost overlaps with that of the first cycle after 35 tensile cycles, indicating that polyAS‐EG_45_ electrolyte has almost no energy loss in multiple cycles, and possesses excellent mechanical fatigue resistance (Figure 3b). Meanwhile, we further investigated the recovery ability of polyAS‐EG_45_ electrolyte at room temperature by rheology. As shown in Figure S6 (Supporting Information) and Figure 3c, the strain is kept at 200% oscillation for 100, 200, and 400 s, respectively, and then immediately recovers to 1% strain. G′ and G″ quickly recover to their original values, even at −50 °C, G′ and G″ still recover to their original state without any loss, which indicates that the polyAS‐EG_45_ electrolyte possesses excellent recovery ability.

In addition to good mechanical strength, the polar and zwitterionic group of polyAS‐EG_45_ electrolyte can enhance the interfacial adhesion between the substrate and the electrolyte. As shown in Figure S7 (Supporting Information), the polyAS‐EG_45_ electrolyte can be adhered to the surface of various materials, including metal, glass, plastic and PTFE. The adhesion strength of polyAS‐EG_45_ electrolyte to carbon cloth can reach ≈180 N m^−1^ (Figure 3d), which is much higher than that of the electrolyte reported in our previous work^[^ ^49^ ^]^ and others' work^[^ ^50^ ^]^ and can provide sufficient stickiness to bind two electrodes together. In addition, the polyAS‐EG_45_ electrolyte shows excellent water retention ability and still has 81% water retention after seven days of exposure to air (Figure S8, Supporting Information).

The polyAS‐EG_45_ electrolyte also has excellent resistance response. As shown in Figure 3e, one end of the polyAS‐EG_45_ is fixed, and then the other end is pulled to change its strain and record its resistance variation. Different strains show different resistance responsiveness. When the strain is kept constant, the resistance remains unchanged accordingly, indicating the stable internal structure of the electrolyte. More importantly, due to the excellent freezing resistance, the electrolyte also shows a stable resistance change when the ambient temperature is below zero (Figure 3f), suggesting that the hydrogel electrolyte has the potential to act as a temperature sensor even at extreme low temperatures, unlike most reported hydrogel sensors only operating above 0 °C.^[^ ^51^, ^52^ ^]^

**Figure 4:**
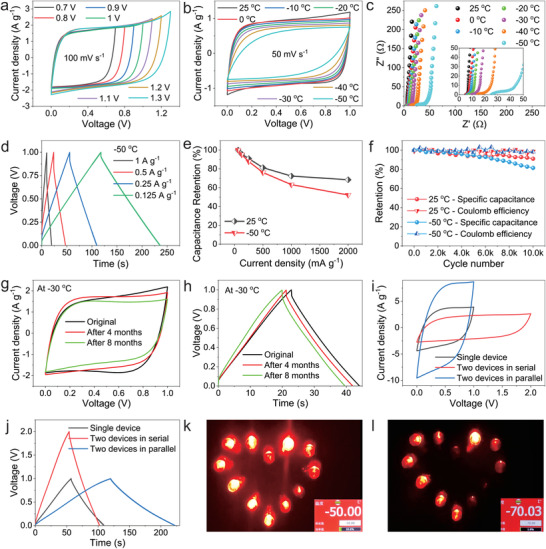
The electrochemical performance of polyAS‐EG_45_ electrolytes‐based SC. a) The voltage window of the assembled SC. b) CV curves of the SC at different temperatures. c) EIS curves of the SC at different temperatures. d) GCD curves of the SC at different current densities at −50 °C. e) Capacitance retention of the SC at 25 and −50 °C. f) The specific capacitance retention and coulomb efficiency of the SC after 10 000 cycles. g) CV and h) GCD curves of the SC remaining at −30 °C for 4 and 8 months. i) CV and j) GCD curves of two SCs connected in serial and in parallel. The photograph of four SCs connected in serial lighting up 12 LED lights at k) −50 °C and l) −70 °C.

Next, the polyAS‐EG_45_ electrolyte is assembled into a SC utilizing carbon cloth with active carbon as electrode. The hydrogel electrolyte with a thickness of about 0.2 mm can directly replace liquid electrolyte and separator, avoiding electrolyte leakage (Figure S9, Supporting Information). Firstly, the voltage windows of SCs were tested at a scanning speed of 100 mV s^–1^. At the electrochemical window of 1 V, the cyclic voltammetry (CV) curves of SC show an almost perfect rectangle (**Figure** **4**a). Thus, the electrochemical window of 1 V is fixed. The CV curves of SC at different scanning speeds of 10–500 mV s^–1^ behave rectangular in shape at room temperatures. The galvanostatic charge–discharge (GCD) curves at different current densities also show a standard triangle and negligible voltage drop, indicating that the SC presents an ideal electric double‐layer capacitance behavior at room temperatures (Figure S10, Supporting Information).

When the temperature is decreased to subzero temperature, the CV curves gradually changes from rectangular shape at 25 °C to slightly deformed rectangular shape at −50 °C (Figure 4b). The GCD curves at different temperatures show the almost triangle shape (Figure S11, Supporting Information), indicating the good electrical behavior. The electrochemical impedance spectroscopy (EIS) at different temperatures shows that the resistance of SC increases from 4.9 Ω at 25 °C to 25 Ω at −50 °C (Figure 4c). In the low frequency region, the EIS curve is almost perpendicular to the X axis, which reflects the fast ion diffusion behavior inside the polyAS‐EG_45_ electrolyte. At −50 °C, the SC still shows good rate performance as confirmed by CV (Figure S12, Supporting Information) and GCD curves (Figure 4d). The mass specific capacitance of SC calculated by GCD curves is shown in Figure S13 (Supporting Information). At 25 °C, the mass specific capacitance is 93.5 F g^−1^ at 62.5 mA g^−1^ and retains 68.5% to 64.0 F g^−1^ at 2 A g^−1^ (Figure 4e, Figure S13, Supporting Information). When the temperature decreases to −50 °C, the mass specific capacitance changes to 62.0 F g^−1^ at 62.5 mA g^−1^ (66.3% of that at 25 °C) and retains 52.3% at 2 A g^−1^, indicating the good electrochemical performance at extreme low temperature.

The cycle stability of SC is also a performance index in practical use. As shown in Figure S14 (Supporting Information), at 25 °C after the SC is charged and discharged for 10 000 times at a current density of 1 A g^−1^, the area of CV curves and the discharging time is slightly smaller than that of the first cycle. The capacitance retention of the SC can reach 91.1% and the coulomb efficiency can be maintained at almost 100% after 10 000 cycles (Figure 4f). When the temperature drops to −50 °C, although the CV and GCD curves are slightly changes after 10 000 cycles (Figure S15, Supporting Information), the capacitance retention of the SC can still reach more than 81.5% and the coulomb efficiency can still be close to 100% (Figure 4f), demonstrating that the SC possesses good cyclic stability in extreme low temperature environment. Furthermore, we also compared the performance of the polyAS‐EG_45_ assembled SC with that of other antifreezing electrolyte assembled SC. As shown in Table S3 (Supporting Information), our SC exhibits excellent frost resistance and low temperature cycle stability among the recent reported devices. More importantly, the polyAS‐EG_45_ assembled SC was stored at −30 °C to test its long‐term low temperature resistance. As shown in Figure 4g,h, the area of the CV curves slightly changes and the charge–discharge time decreases a little after four months of storage at −30 °C. Even stored at −30 °C for eight months, the SC can still work normally and the capacitance can only reduce to 92.0% of the original one, indicating that the polyAS‐EG_45_ electrolyte has the ability to work at low temperature in a long term. In practical applications, higher voltage and higher energy density can be obtained by connecting several SCs in parallel and in series. In our case, compared with a single SC, two SCs in series show an electrochemical window of 2 V in both CV and GCD test; On the other hand, the current density of two SCs in parallel increases to twice that of a single SC (Figure 4i,j). In addition, four SCs assembled in serial can light up 12 LED lamps for more than 10 min at −50 °C (Figure 4k, Movie S1, Supporting Information). Even if the temperature decreases to −70 °C, only the brightness of the LED lamps has dimmed and can still last for 10 min (Figure 4l, Movie S2, Supporting Information).

## 3 Conclusion

In short, to solve the freezing resistance of hydrogel electrolyte in subzero temperature, we have fabricated a serial of proton hydrogel electrolytes by random copolymerization of AM and SBMA in the presence of 1 m H_2_SO_4_ and EG. The ionic bonds and the hydrogen bonds in the system provide the mechanical strength and antifreezing ability. An extreme low‐temperature conductivity of 1.51 mS cm^–1^ at −50 °C was obtained. FTIR, Raman and BDS as well as molecular dynamic demonstrate that the proton transfer is facilitated by hopping on the zwitterionic group and Grotthuss proton conductivity mechanism is dominated at glassy state of the polymer chains. The electrolyte‐assembled SC offers high specific capacitance of 93.5 F g^–1^ at 25 °C and 62 F g^–1^ at −50 °C with a capacitance retention of 91.1% and 81.5% after 10 000 cycles, respectively. The SC can work at −30 °C for eight months with 92.0% capacitance of the original one. This work suggests that our electrolytes are promising candidates for stable and high‐performance energy storage devices that can be used in ultra‐low temperature environments.

## 4 Experimental Section

### Materials

Acrylamide (AM), [(methacryloyloxy)ethyl]dimethyl‐(3‐sulfopropyl)ammonium hydroxide (SBMA), *N*,*N*‐methylenebisacrylamide (MBA), and ammonium persulfate (APS) were purchased from Aladdin. Ethylene glycol (EG) was purchased from Sinopharm. Poly(vinylidene fluoride) (PVDF) and 1‐methy l‐2‐pyrrolidone (NMP) were supplied by Macklin. Carbon cloth was purchased from Cetech, Taiwan. Activated carbon (AC, YP‐50F) was purchased from Kuraray Chemical, Japan. Carbon black was purchased from Alfa Aesar.

### Preparation of Antifreezing Zwitterionic PolyAS‐EG Electrolyte

The polyAS‐EG gel electrolyte was obtained by random copolymerization of AM and SBMA. First, AM and SBMA with different molar ratio (total mass: 2 g) were added to EG and H_2_O mixture (EG: 0, 30, 45, 60, 100 vol% of the volume of EG/H_2_O mixture) with 1 m H_2_SO_4_ and 0.2 mg mL^–1^ of MBA, followed by stirring for 1 h in an ice bath. Subsequently, 0.02 g of initiator APS (1 wt% relative to the total mass of monomers) was added to the above solution and stirred for 30 min under ice bath stirring. Finally, the resulting precursor solution was sonicated for 10 min to remove air bubbles and the precursor solution was injected into a mold, sealed and polymerized at 45 °C for 12 h.

### Assembly of Supercapacitor (SC) Devices

First, 80 mg of AC, 10 mg of conductive carbon black and 10 mg of PVDF were ground and an appropriate amount of NMP was added to form a homogeneous dispersion. Then the dispersion was uniformly applied on carbon cloth and placed in a vacuum oven at 180 °C for 24 h to obtain AC electrodes. The mass of active material on each electrode was recorded. Two AC electrodes with the same mass of loaded active material were covered on both sides of the polyAS‐EG_45_ hydrogel electrolyte to produce a “sandwich structure” of the solid SC.

## Conflict of Interest

The authors declare no conflict of interest.

## Supporting information

Supporting Information

Supplemental Movie 1

Supplemental Movie 2

## Data Availability

The data that support the findings of this study are available from the corresponding author upon reasonable request.
