# Supplementary material for: Antifreezing Proton Zwitterionic Hydrogel Electrolyte via Ionic Hopping and Grotthuss Transport Mechanism toward Solid Supercapacitor Working at −50 °C
Source: Adv Sci (Weinh). 2022 Jul 26;9(27):2201679. doi: 10.1002/advs.202201679 (PMC9507348; doi:10.1002/advs.202201679)
Supplement: Supplementary file 1 — Supporting Information [file ADVS-9-2201679-s002.pdf]

ADVANCED SCIENCE
Open Access

Supporting Information

for *Adv. Sci.*, DOI 10.1002/advs.202201679

Antifreezing Proton Zwitterionic Hydrogel Electrolyte via Ionic Hopping and Grotthuss Transport Mechanism toward Solid Supercapacitor Working at −50 °C

*Weigang Sun, Zhen Xu, Congde Qiao, Bingxi Lv, Ligang Gai, Xingxiang Ji, Haihui Jiang and Libin Liu**

Supporting Information

Antifreezing proton zwitterionic hydrogel electrolyte via ionic hopping and Grotthuss transport mechanism towards solid supercapacitor working at −50 °C

Weigang Sun, Zhen Xu, Congde Qiao, Bingxi Lv, Ligang Gai, Xingxiang Ji, Haihui Jiang, Libin Liu*

School of Chemistry and Chemical Engineering, State Key Laboratory of Biobased Material and Green Papermaking, Qilu University of Technology (Shandong Academy of Sciences), Jinan 250353, China

**Corresponding author. E-mail: lbliu@qlu.edu.cn* (Libin Liu)

Experimental Section

Rheology

Rheological measurements were performed with an ARES-G2 rheometer using a parallel plate with a diameter of 25 mm. Frequency sweep was tested at a fixed 1% oscillatory strain in the frequency range of 0.1~100 rad s^{-1} at 25 ^{o}C and -50 ^{o}C, respectively. The temperature scan was performed at a fixed frequency of 6.28 rad s^{-1} in the temperature range of -80 to 60 ^{o}C. Alternating step strain (1 and 200%) scans of the electrolyte were recorded at a constant angular frequency of 10 rad s^{-1}.

Mechanical Testing

Mechanical measurements were performed with a general test apparatus (Hensgrand, WDW-02, China). The electrolyte stretching test was performed at room temperature by stretching a 6 mm diameter and 40 mm length cylinder at a stretching speed of 100 mm min^{-1}. The stretching cycle was performed by fixing the stain of 600% with an interval of 10 min after each cycle.

The T-peel test is performed at room temperature. One substrate was held in place and the other was peeled off with a stretching speed of 100 mm min^{-1}. The coverage area is 4 mm x 100 mm.

Water retention testing of zwitterionic gel electrolytes

Two pieces of polyAS-EG_0 and polyAS-EG_{45} gel electrolytes of the same mass were taken, exposed to air at room temperature, and their masses were recorded every 12 hours.

Broadband Dielectric Spectroscopy (BDS)

Broadband dielectric measurements were performed over a frequency range of 10^{-2}~10^7 Hz using the Novocontrol Concept 80 system. The electrolytes were placed between two electrode plates and their dielectric data were measured at ambient temperatures of -30, -50 and -70 ^{o}C, respectively. The electrolytes were equilibrated at each temperature for 30 min prior to the dielectric measurements.

Conductivity measurement of zwitterionic hydrogel electrolytes

The ionic conductivity (σ) of the hydrogel electrolytes was measured on a CHI 660E electrochemical workstation using a dual-probe method. The zwitterionic hydrogel electrolytes were fixed between two stainless steel fixtures and the conductivity was measured at the corresponding temperature after stabilization at different temperatures for 1~2 hours. Each group of samples was measured 3~5 times and the average value was taken. The ionic conductivity was calculated according to equation:

$$\sigma = \frac{L}{RS}$$

where R is the resistance (Ω), S is the cross-sectional area of the electrolyte under test (cm^2), and L is the thickness of the tested electrolyte (cm).

Measurement of electrochemical performance of SC devices

Cyclic voltammetry (CV), electrochemical impedance spectroscopy (EIS), and electrostatic charging and discharging (GCD) measurements were performed in a two-electrode system using a CHI 660E workstation. CV was performed at different scan rates between 0 and 1 V. EIS was performed at amplitudes of 10 mV between 0.01 Hz ~100 kHz. GCD was performed at different current densities between 0~1 V. Cycling stability and coulomb efficiency were measured by 10,000 GCD cycles. The mass specific capacitance of an electrode (C_{sp}, F g^{-1}) is obtained from the electrostatic discharge curve and is calculated according to the following equation.

$$C_{sp} = 4\ \frac{I\Delta t}{m_{device}\Delta V}$$

where I (mA) is the discharge current, Δt (s) is the discharge time, m_{device} (mg) is the total mass of the capacitor electrodes, and ΔV (V) represents the discharge voltage.

Density functional theory (DFT) calculations

The 7 structures (H_2O, EG, SBMA, AM, H^+, SO_4^{2-} , HSO_4^-) and their 20 complexes were first optimized by using the density functional theory (DFT) at the B3LYP/def2-SVP level.[1] All geometry optimizations including the solvent effect with PCM[2] were performed using the Gaussian 09 package, revision B. 01.[3] Then the single-point energies of four complexes were done at the same level after previous optimization, which considering basis set superposition error (BSSE). The harmonic frequency calculations were carried out at the same level of theory to help verify that all structures have no imaginary frequency.

The binding energy of the configuration (E_{bind}) was calculated by the following equation:

$$E_b = E_{AB} - (E_A + E_B)$$

Where E_A , E_B and E_{AB} respectively represent the energies of A and B (every single structure) and the total energy , a negative value of Eb indicates that the process is an

exothermic reaction and high negative value corresponds to a stronger interaction, which indicates more heat release and a more stable product.

Molecular dynamics (MD)

The model was built by Amorphous Cell modules of Materials Studio software, model 1 molar ratio of each component was AM : SBMA : H_2O : glycol : H_2SO_4 = 3 : 1 : 38 : 10 : 1, and model 2 was AM : H_2O : glycol : H_2SO_4 = 3 : 38 : 10 : 1. Molecular dynamics (MD) simulations were carried out with Forcite Plus of Materials Studio software (Biovia Inc.). The temperature was controlled at 203 K and 298 K by a Nose-Hoover thermostat. In order to obtain a reasonable density, the dynamics of 100 ps was carried out with NPT ensemble. Then canonical ensemble (NVT) was applied for each system using the velocity Verlet algorithm, and the time step was set as 1 fs. Van der Waals interactions were calculated by an atom-based method with a cutoff distance of 12.5 Å. Electrostatic interactions were calculated by the Ewald method, which takes a long time but is accurate for long-range interactions. Finally, each system was simulated for 500 ps to reach equilibrium state. After simulation, we analyzed the stability of foam systems through their equilibrium configurations and dynamic process.

The mean square displacements (MSD) calculated from equation:

$$MSD(t) = \left\{ \frac{1}{N} \sum_{i=1}^{N} |r_i(t) - r_i(0)|^2 \right\}$$

where N is the number of target molecules and $r_i(t)$ is the position of molecule i at time t. The self-diffusion coefficient represents the mobility of the transference for the molecules. Diffusion coefficients (D) can then be obtained from the slope of the mean square displacement versus time curve, using the well-known Einstein relation:

$$D_\alpha = \frac{1}{6N_\alpha} \lim_{t \to \infty} \frac{d}{dt} \sum_{i=1}^{N_\alpha} \{[r_i(t) - r_i(0)]^2\}$$

where d is the dimensionality of the system, r_i *(t)* and r_i *(0)* are the center-of-mass coordinates of the ith water molecule at times t and $t = 0$, respectively.

Other Characterization

Raman spectra were recorded with a LabRAM tHR800 Raman spectrometer (HORIBA JY, France) with an excitation wavelength of 532 nm. Differential scanning calorimetry (DSC) was tested with a TA2500 instrument by first lowering the sample from room temperature to -80 ^{o}C and then raising the sample from -80 ^{o}C to 40 ^{o}C. The cooling and heating rates were both 2.5 ^{o}C min^{-1}, and the sample masses taken were in the range of 5-10 mg. FTIR was tested with an ENSOR Fourier transform infrared spectrometer (Bruker, USA) was used to perform the tests.

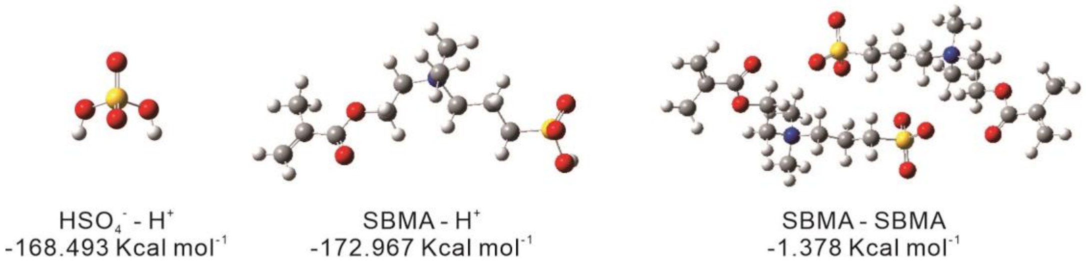

Figure S1. The interaction energy of HSO_4^--H^+, SBMA-H^+ and SBMA-SBMA.

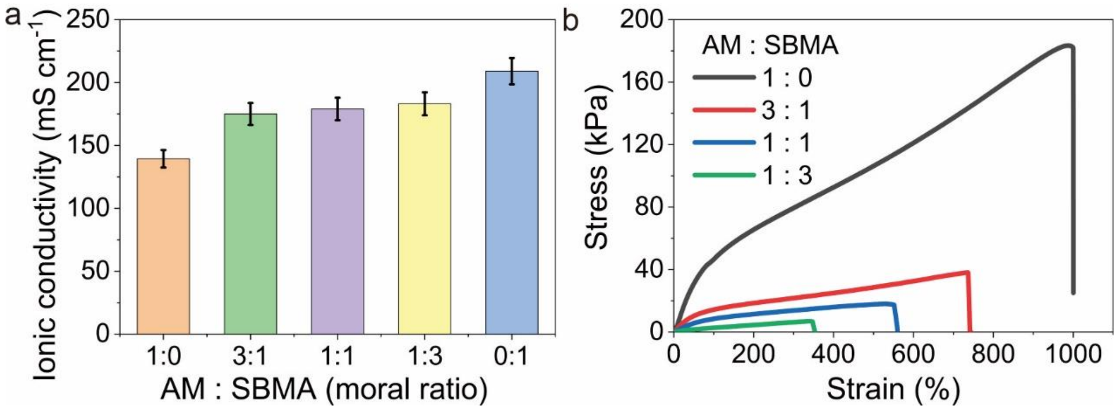

Figure S2. The molar ratio of AM and SBMA effect on the ionic conductivity (a) and mechanical strength (b) at room temperature.

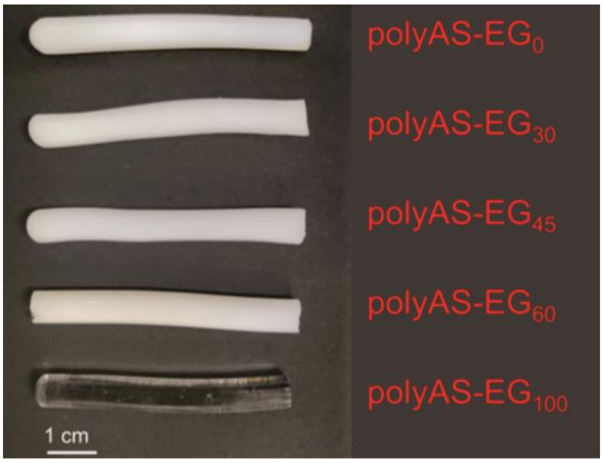

Figure S3. Photographs of gel electrolytes with different EG contents.

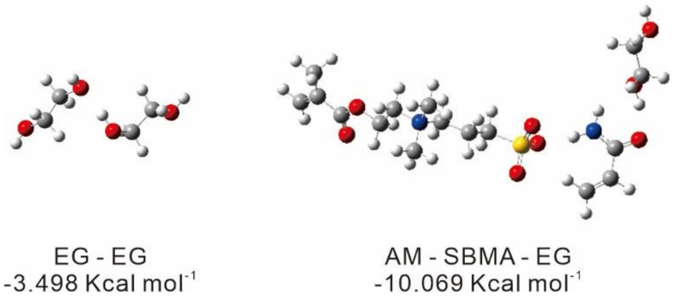

Figure S4. The interaction energy of EG-EG and AM-SBMA-EG.

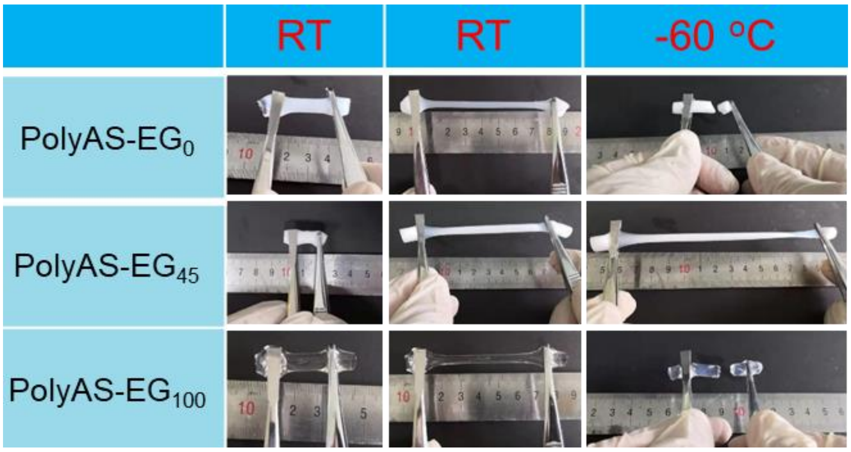

Figure S5. The photographs of different electrolytes at different temperatures.

Table S1. Diffusion coefficients of H_3O^+ in polyAS and polyAS-EG_{45} from their MSDs at different temperatures.

System	Value (10^{-6} cm^2 s^{-1})
PolyAM at 25 ^oC	1.745

PolyAS-EG_{45} at 25 °C	3.082
PolyAM at -70 °C	0.138
PolyAS-EG_{45} at -70 °C	0.160

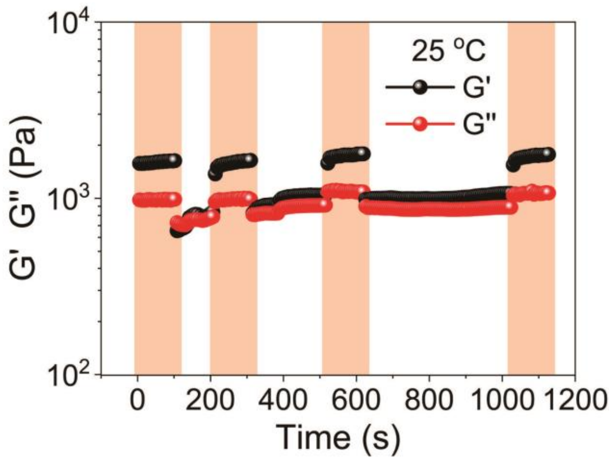

Figure S6. G' and G" of the polyAS-EG_{45} electrolytes at 25 °C under an alternate strain of 1% for 100 s and 200% for 100, 200 and 400 s, respectively.

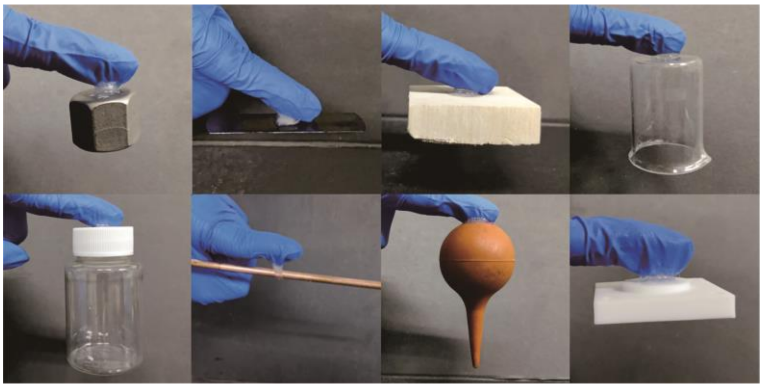

Figure S7. Photograph of polyAS-EG_{45} electrolyte adhering to iron, silicon, wood, glass, plastic, copper rod, rubber and PTFE.

Table S2. Comparison of low temperature conductivity and mechanical properties of

polyAS-EG_{45} electrolyte with that of published literatures.

Conductive media (concentration)	Antifreeze	Water Retention	Strain (%)	Stress (kPa)	Ionic conductivity (mS cm^{-1})	Refs
H_2SO_4(1M)	EG	81%	~1390.1	~21.3	1.51 at -50 ^oC	Our work
H_2SO_4(1M)	EG	90%	N/A	N/A	4.8 at -40 ^oC	[4]
H_2SO_4(2M)	DMSO	N/A	~22.6	~1.43 x 10^4	0.017 at -50 ^oC	[5]
KOH(6M)	KOH	82.64%	~1400	N/A	57 at -20 ^oC	[6]
KOH(5M)	KOH	N/A	~170	~27	18.1 at -20 ^oC	[7]
NaOH(0.01mol)	EG	N/A	~900	~60	14.6 at -20 ^oC	[8]
LiCl(7M)	LiCl	66%	~270	~5	12.6 at -40 ^oC	[9]
LiTFSI(3M)	LiTFSI	N/A	~305	~28	0.1 at -40 ^oC	[10]
LiCl(1.1M)	EG	N/A	~300	~100	2.38 at -40 ^oC	[11]
$ZnSO_4$(2M) /$MnSO_4$(0.2M)	Glycerol	N/A	~502	~115.5	10.1 at -35 ^oC	[12]
$CaCl_2$(30 wt%)	Glycerol	68.90%	~793	~530	10 at RT	[13]
KCl(2M)	EMImAc	80%	~97	~360	0.16 at -50 ^oC	[14]
KCl(0.28M)	PIL	N/A	~1050	~20	11 at -20 ^oC	[15]
H_3PO_4(75 vol%)	H_3PO_4	55.86%	~2800	~110	N/A	[16]

Abbreviations: LiTFSI (lithium bis(trifluoromethanesulfonyl)imide); EG (Ethylene glycol); DMSO (Dimethyl sulfoxide); EMImAc (1-ethyl-3-methylimidazolium acetate); PIL (copolymerization of 1-vinyl-3-(carboxymethyl)-imidazole (zwitterionic ionic liquid) and acrylamide (AAm)).

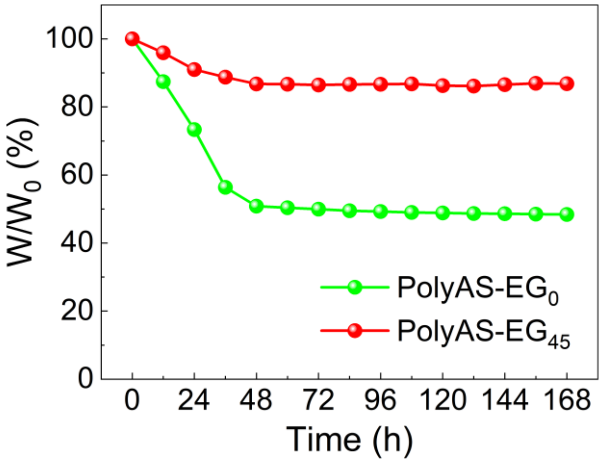

Figure S8. Weight variation of the polyAS-EG_0 and polyAS-EG_{45} electrolyte at room temperature.

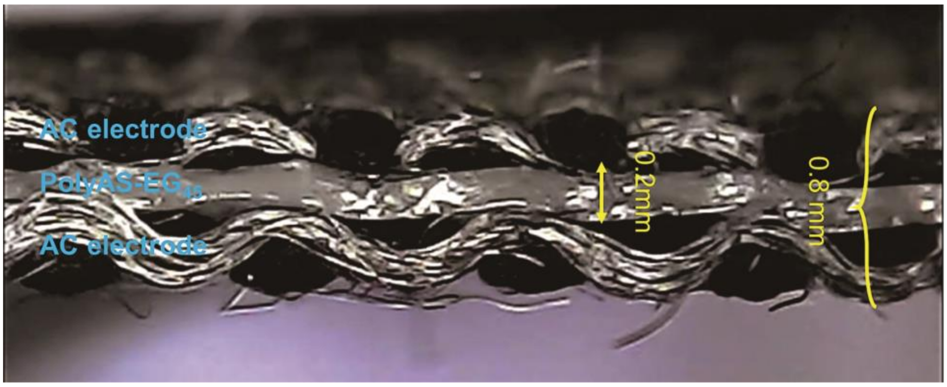

Figure S9. Photograph of polyAS-EG_{45} electrolyte assembled SC using active carbon electrode.

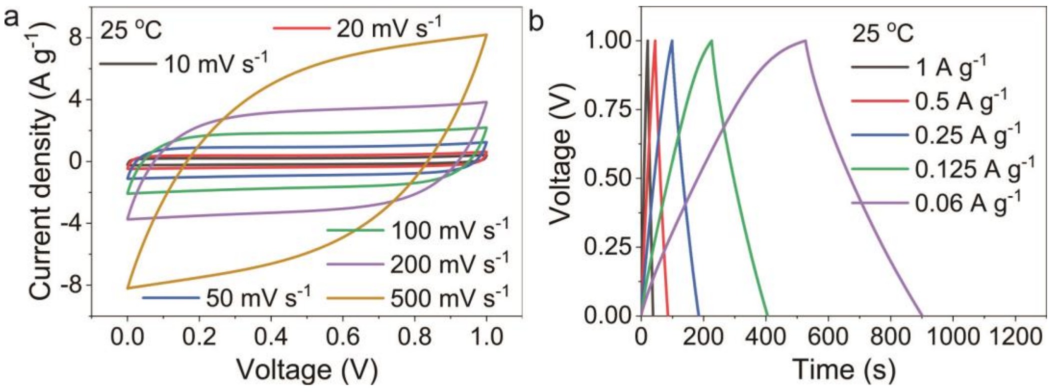

Figure S10. (a) CV curves of the SC at different scan rates at 25 ^oC. (b) GCD curves of the SC at different current density at 25 ^oC.

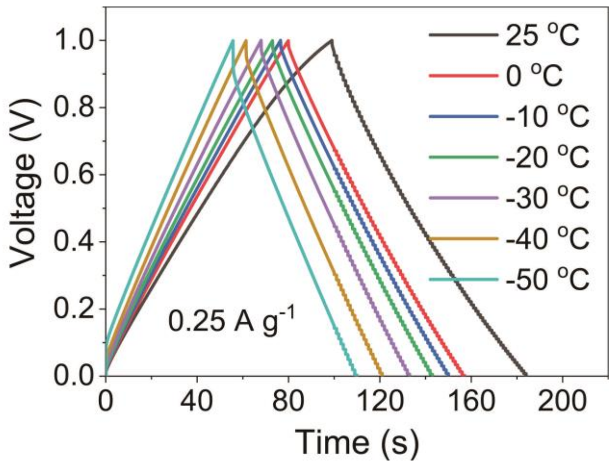

Figure S11. GCD curves of the SC at different temperatures.

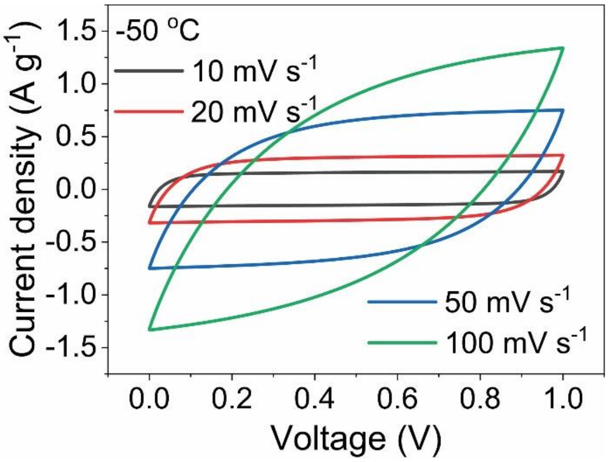

Figure S12. CV curves of the SC at different scan rates at -50 ^{o}C.

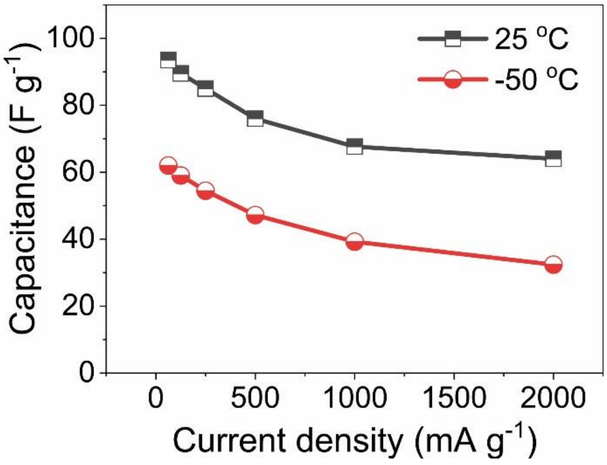

Figure S13. The mass specific capacitance of the SC at 25 and -50 ^{o}C.

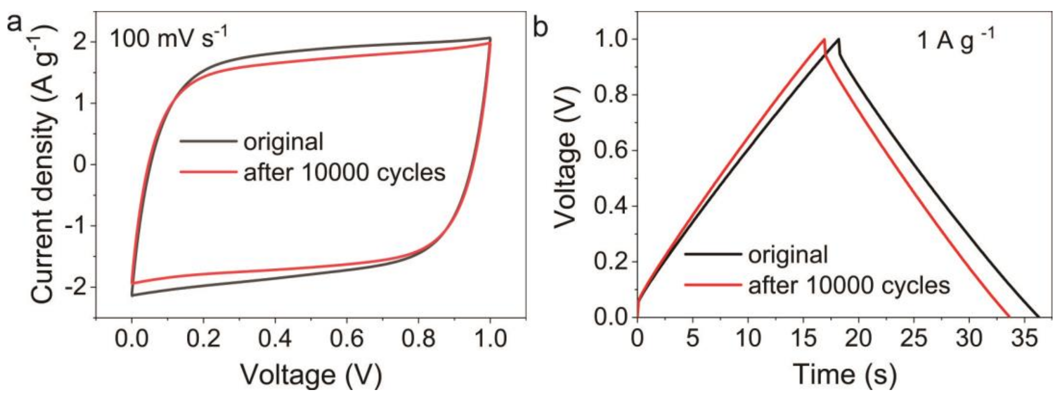

Figure S14. (a) CV and (b) GCD curves of the SC after10,000 cycles at 25 °C.

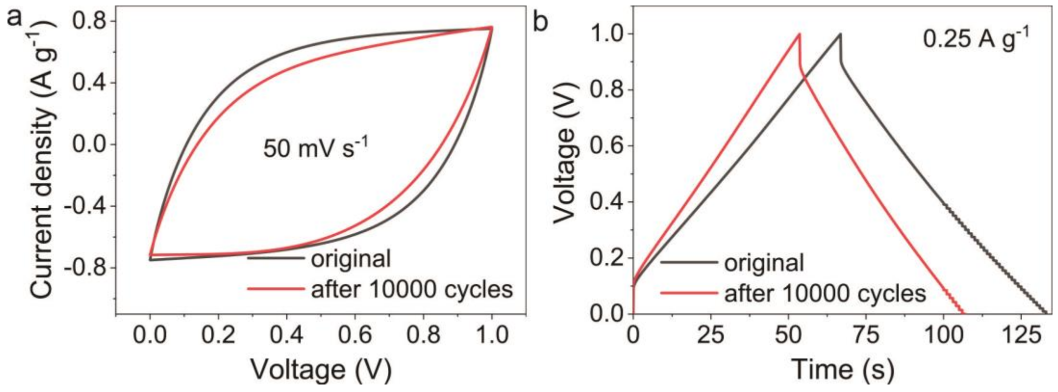

Figure S15. (a) CV and (b) GCD curves of the SC after10,000 cycles at -50 °C.

Table S3. Comparison of the low temperature performance of the supercapacitor assembled by polyAS-EG_{45} electrolyte with that of published literatures.

Electrodes	Electrolytes	Capacitance retention (vs RT)	Cycle stability (cycle number/°C)	Working Temperature	Refs
AC	polyAS-EG_{45}	66.3% at -50 °C	81.5% (10,000 at -50 °C)	-70 °C	Our work
AC	polySH-7	78% at -30 °C	71% (10,000 at -30 °C)	-30 °C	[9]
AC	poly($HFBA_8$-co-$HEMA_1$)	N/A	79% (8,000 at -20 °C)	RT	[10]
AC	CS-PAAm	N/A	97.5% (10,000 at RT)	N/A	[16]
AC	SPGC	N/A	N/A	N/A	[13]
CNT fibers	SF/EMImAc	76% at -50 °C	99.3% (1,000 at -50 °C)	N/A	[14]
CNT paper	PVA	70.6% at -40 °C	88.3% (5,000 at -20 °C)	N/A	[11]
Graphene	MMT/PVA	57% at -40 °C	95% (10,000 at RT)	Cold ice	[5]
PANI/Carbon cloth	PVA-MA/AM	81% at -40 °C	N/A	N/A	[4]
α-MnO_2/CNT-Zn	EG-waPUA/PAM	N/A	N/A	-20 °C	[8]
NiCo/Zn	PANa	N/A	N/A	-20 °C	[6]
Zn/Carbon cloth	PAMPS-K/MC	N/A	N/A	-20 °C	[7]

Abbreviations: AC (Activated carbon); CNT fibers (carbon nanotube fibers); CNT paper (carbon nanotube paper); PANI (polyaniline); polySH-7 : poly[2-(methacryloyloxy)ethyl]dimethyl-(3-sulfopropyl)ammonium hydroxide-co-2-hydroxyethyl acrylate] with 7M LiCl; poly(HFBA8-co-HEMA1) (Copolymerization of hexafluorobutyl acrylate (HFBA) and hydroxyethyl methacrylate (HEMA) in a molar ratio of 8:1); CS-PAAm (CS-PAAm hydrogel obtained by copolymerization of chitosan and acrylamide.); SPGC (Starch, PVA, glycerol and $CaCl_2$); SF/EMImAc (SF/EMImAc hydrogel electrolytes were prepared from silk fibroin (SF)/1-ethyl-3-methylimidazole acetate (EMImAc).); PVA (polyvinyl alcohol); MMT/PVA (montmorillonite/polyvinyl alcohol); PVA-MA/AM (Methacrylate functionalized polyvinyl alcohol (PVA-MA)/acrylamide (AM));

EG-waPUA/PAM (EG-based waterborne anionic polyurethane acrylates/polyacrylamide); PANa (Sodium polyacrylate hydrogel); PAMPS-K/MC (poly(2-acrylamido-2-methylpropanesulfonic acid potassium salt)/methyl cellulose).

References

[1] W. Kohn, L. J. Sham, Phys. Rev. **1965**, 140, A1133.

[2] S. Miertuš, E. Scrocco, J. Tomasi, Chem. Phys. **1981**, 55, 117.

[3] Gaussian 09, Revision A.02, M. J. Frisch, G. W. Trucks, H. B. Schlegel, G. E. Scuseria, M. A. Robb, J. R. Cheeseman, G. Scalmani, V. Barone, G. A. Petersson, H. Nakatsuji, X. Li, M. Caricato, A. Marenich, J. Bloino, B. G. Janesko, R. Gomperts, B. Mennucci, H. P. Hratchian, J. V. Ortiz, A. F. Izmaylov, J. L. Sonnenberg, D. Williams-Young, F. Ding, F. Lipparini, F. Egidi, J. Goings, B. Peng, A. Petrone, T. Henderson, D. Ranasinghe, V. G. Zakrzewski, J. Gao, N. Rega, G. Zheng, W. Liang, M. Hada, M. Ehara, K. Toyota, R. Fukuda, J. Hasegawa, M. Ishida, T. Nakajima, Y. Honda, O. Kitao, H. Nakai, T. Vreven, K. Throssell, J. A. Montgomery, Jr., J. E. Peralta, F. Ogliaro, M. Bearpark, J. J. Heyd, E. Brothers, K. N. Kudin, V. N. Staroverov, T. Keith, R. Kobayashi, J. Normand, K. Raghavachari, A. Rendell, J. C. Burant, S. S. Iyengar, J. Tomasi, M. Cossi, J. M. Millam, M. Klene, C. Adamo, R. Cammi, J. W. Ochterski, R. L. Martin, K. Morokuma, O. Farkas, J. B. Foresman, and D. J. Fox, Gaussian, Inc., Wallingford CT, 2016.

[4] Z. Liu, J. Zhang, J. Liu, Y. Long, L. Fang, Q. Wang, T. Liu, J. Mater. Chem. A 2020, 8, 6219.

[5] Chao Lu, Xi Chen, Nano Lett. **2020**, 20, 1907.

[6] H. Wang, J. Liu, J. Wang, M. Hu, Y. Feng, P. Wang, Y. Wang, N. Nie, J. Zhang, H. Chen, Q. Yuan, J. Wu, Y. Huang, ACS Appl. Mater. Interfaces **2019**, 11, 49.

[7] N. Sun, F. Lu, Y. Yu, L. Su, X. Gao, L. Zheng, ACS Appl. Mater. Interfaces **2020**, 12, 11778.

[8] F. Mo, G. Liang, Q. Meng, Z. Liu, H. Li, J. Fan, C. Zhi, Energy Environ. Sci.

2019, 12, 706.

[9] J. Yang, Z. Xu, J. Wang, L. Gai, X. Ji, H. Jiang, Li. Liu, Adv. Funct. Mater. **2021**, 31, 2009438.

[10] J. Wang, X. Li, J. Yang, W. Sun, Q. Ban, L. Gai, Y. Gong, Z. Xu, L. Liu, ChemSusChem **2021**, 14, 2056.

[11] Q. Rong, W. Lei, J. Huang, and M. Liu, Adv. Energy Mater. **2018**, 8, 1801967.

[12] M. Chen, W. Zhou, A. Wang, A. Huang, J. Chen, J. Xu, C.-P. Wong, J. Mater. Chem. A **2020**, 8, 6828.

[13] J. Lu, J. Gu, O. Hu, Y. Fu, D. Ye, X. Zhang, Y. Zheng, L. Hou, H. Liu, X. Jiang, J. Mater. Chem. A **2021**, 9, 18406.

[14] W. Wang, Y. Liu, S. Wang, X. Fu, T. Zhao, X. Chen, Z. Shao, ACS Appl. Mater. Interfaces **2020**, 12, 25353.

[15] Z. Liu, Y. Wang, Y. Ren, G. Jin, C. Zhang, W. Chen, F. Yan, Mater. Horiz. **2020**, 7, 919.

[16] J. Xu, R. Jin, X. Ren, G. Gao, Chem. Eng. J. **2021**, 413, 127446.
